# Use Characteristics and Triage Acuity of a Digital Symptom Checker in a Large Integrated Health System: Population-Based Descriptive Study

**DOI:** 10.2196/20549

**Published:** 2020-11-30

**Authors:** Keith E Morse, Nicolai P Ostberg, Veena G Jones, Albert S Chan

**Affiliations:** 1 Department of Pediatrics Stanford University School of Medicine Palo Alto, CA United States; 2 Center for Biomedical Informatics Research Stanford University School of Medicine Palo Alto, CA United States; 3 Clinical Leadership Team Sutter Health Sacramento, CA United States; 4 Palo Alto Medical Foundation Research Institute Palo Alto, CA United States

**Keywords:** symptom checker, chatbot, computer-assisted diagnosis, diagnostic self-evaluation, artificial intelligence, self-care, COVID-19

## Abstract

**Background:**

Pressure on the US health care system has been increasing due to a combination of aging populations, rising health care expenditures, and most recently, the COVID-19 pandemic. Responses to this pressure are hindered in part by reliance on a limited supply of highly trained health care professionals, creating a need for scalable technological solutions. Digital symptom checkers are artificial intelligence–supported software tools that use a conversational “chatbot” format to support rapid diagnosis and consistent triage. The COVID-19 pandemic has brought new attention to these tools due to the need to avoid face-to-face contact and preserve urgent care capacity. However, evidence-based deployment of these chatbots requires an understanding of user demographics and associated triage recommendations generated by a large general population.

**Objective:**

In this study, we evaluate the user demographics and levels of triage acuity provided by a symptom checker chatbot deployed in partnership with a large integrated health system in the United States.

**Methods:**

This population-based descriptive study included all web-based symptom assessments completed on the website and patient portal of the Sutter Health system (24 hospitals in Northern California) from April 24, 2019, to February 1, 2020. User demographics were compared to relevant US Census population data.

**Results:**

A total of 26,646 symptom assessments were completed during the study period. Most assessments (17,816/26,646, 66.9%) were completed by female users. The mean user age was 34.3 years (SD 14.4 years), compared to a median age of 37.3 years of the general population. The most common initial symptom was abdominal pain (2060/26,646, 7.7%). A substantial number of assessments (12,357/26,646, 46.4%) were completed outside of typical physician office hours. Most users were advised to seek medical care on the same day (7299/26,646, 27.4%) or within 2-3 days (6301/26,646, 23.6%). Over a quarter of the assessments indicated a high degree of urgency (7723/26,646, 29.0%).

**Conclusions:**

Users of the symptom checker chatbot were broadly representative of our patient population, although they skewed toward younger and female users. The triage recommendations were comparable to those of nurse-staffed telephone triage lines. Although the emergence of COVID-19 has increased the interest in remote medical assessment tools, it is important to take an evidence-based approach to their deployment.

## Introduction

Health care services in the United States are facing increasing levels of pressure, driven by a combination of aging populations, economic reform of health services, and more recently, the emergence of the COVID-19 pandemic [1].Training health care professionals is a slow process, and with widespread shortages of trained personnel and key vacancies throughout the system [2], scalable technological alternatives must be evaluated. One potential approach is a digital symptom checker, which is an artificial intelligence (AI)–supported software tool that uses a conversational “chatbot” format to ask questions about a patient’s symptoms and returns a list of likely diagnoses to support self-diagnosis and appropriate triage [3].

The COVID-19 pandemic has brought new urgency to the consideration of chatbots due to the need to avoid face-to-face contact, preserve in-person care capacity, and triage patients at unprecedented volumes [4]. However, digital tools that impact care delivery should undergo rigorous evaluation that enables evidence-based determination of their efficacy. Symptom checker triage recommendations have been theorized to reduce unnecessary clinic and emergency room visits [5], and a recent study showed that completing a web-based symptom assessment reduced the urgency of the care that patients intended to seek [6]. However, little is known about aggregate triage recommendations generated by a symptom checker used in larger populations, and a number of recent reviews have called for more research to be shared [5,7,8]. Here, we describe the use characteristics and triage recommendations of one symptom checker chatbot deployed in partnership with a large, integrated health care system in Northern California.

## Methods

### Recruitment

**Figure 1:**
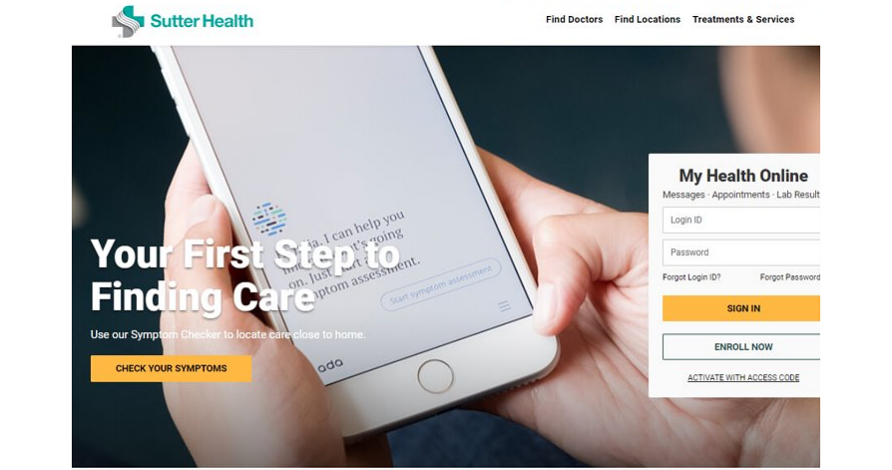
Screenshot of the Sutter Health webpage during the symptom checker launch, May 2019.

The setting for this study is Sutter Health, a not-for-profit health care system in Northern California with 24 hospitals. In 2019, the symptom checker chatbot was introduced across the health system for broad use by any current and prospective patients over the age of 16 years. The chatbot was integrated into the main Sutter Health website (Figure 1) and web-based patient portal. Marketing was performed through several channels, including an email campaign to existing patients and social media advertisements.

The data for this study encompass all symptom assessments completed from April 24, 2019, to February 1, 2020. This study was approved by the Sutter Health Institutional Review Board.

### Symptom Checker

The symptom checker, developed by Ada Health (Ada Health, Berlin, Germany [9]), uses a conversational chatbot-style interface to elicit users’ basic demographics and presenting symptoms as well as additional details such as symptom duration and severity. This information is analyzed by an AI algorithm to produce likely diagnoses and associated triage recommendations. The symptom checker assessments are anonymous; thus, recurrent users could not be identified, nor could use be linked to patient data within the electronic health record of the health system.

### Statistical Analysis

Because this is a descriptive service improvement study, we had no falsifiable hypotheses; therefore, we did not undertake a formal power analysis. For comparison with our broader population, we extracted population-level demographics from the US Census Bureau data of Alameda County, one of the largest counties in Northern California served by Sutter Health [10]. Data analyzed included demographic information entered by the patient, initial symptoms reported, time of assessment, and triage advice generated by the symptom checker chatbot. Triage advice took the form of one of eight possible suggestions, which were sorted into low, medium, or high acuity levels. Low acuity included suggestions to manage symptoms at home, seek medical advice in 2-3 weeks, or seek advice from a pharmacy. Medium acuity included suggestions to seek medical advice in 2-3 days or seek medical advice that same day. High acuity included suggestions to seek care within 4 hours, call an ambulance, or seek care in an emergency department.

## Results

### User Demographics and Time of Use

A total of 26,646 symptom assessments were completed during the study period, with no missing data. Most users (17,816/26,646, 66.9%) were female, and the remainder were male (8830/26,646, 33.1%). The comparator population of Alameda County is 50.9% female [10].

The mean age of the users was 34.3 years (SD 14.4 years); examination of subgroups (Table 1) revealed that the users were most commonly aged 30-39 years (7009/26,646, 26.3%). However, a sizable minority of users were in older age brackets; 3531/26,646 (13.3%) were aged 60 years or older. For comparison, the median age in Alameda County is 37.3 years, and 18.4% of the population is aged 60 or over [10].

Slightly less than half of the assessments (12,357/26,646, 46.4%) were completed outside of the typical physician office hours of 9 AM to 6 PM (Table 1). The most commonly reported initial symptom was abdominal pain (2060/26,646, 7.7%). The top 10 most commonly reported initial symptoms are shown in Table 2.

**Table 1:** Demographics and time of day of symptom checker use (N=26,646).

Characteristic		Count (%)
**Gender**		
	Male	8830 (33.1)
	Female	17,816 (66.9)
**Age (years)**		
	<19	863 (3.2)
	20-29	6441 (24.2)
	30-39	7009 (26.3)
	40-49	4663 (17.5)
	50-59	4139 (15.5)
	60-69	2209 (8.3)
	70-79	951 (3.6)
	80-89	247 (0.9)
	90-99	44 (0.2)
	>100	80 (0.3)
**Time of assessment**		
	12 AM to 2:59 AM	1267 (4.8)
	3 AM to 5:59 AM	1143 (4.3)
	6 AM to 8:59 AM	3768 (14.1)
	9 AM to 11:59 AM	5456 (20.5)
	12 PM to 2:59 PM	4890 (18.4)
	3 PM to 5:59 PM	3943 (14.8)
	6 PM to 8:59 PM	3237 (12.2)
	9 PM to 11:59 PM	2942 (11.0)

**Table 2:** The top 10 most common initial symptoms reported in the symptom checker (N=26,646).

Symptom	Count (%)
Abdominal pain	2060 (7.7)
Cough	1537 (5.8)
Headache	1085 (4.1)
Sore throat	897 (3.4)
Dizziness	621 (2.3)
Fatigue	559 (2.0)
Chest pain	534 (2.0)
Lower back pain	528 (2.0)
Diarrhea	466 (1.7)
Painful urination	460 (1.7)

### Triage Urgency

Based on a user’s symptom presentation, the symptom checker chatbot offered eight levels of triage advice, which were grouped into three levels of acuity (Table 3). Among the 26,646 assessments, 5323 (20.0%) directed the user to low acuity care, 13,600 (51.0%) directed the user to medium acuity care, and 7723 (29.0%) directed the user to high acuity care. The most common triage advice was to seek same-day medical care (Table 3).

**Table 3:** Advice and triage acuity levels of the assessments provided by the symptom checker (N=26,646).

Acuity level and advice		Count (%)	
**High**		7723 (29.0)	
	Call an ambulance		1796 (6.7)
	Seek emergency care		3703 (13.9)
	Seek medical advice within 4 hours		2224 (8.3)
**Medium**		13,600 (51.0)	
	Seek medical advice within the same day		7299 (27.4)
	Seek medical advice within 2-3 days		6301 (23.6)
**Low**		5323 (20.0)	
	Seek medical advice from a pharmacy		3433 (12.9)
	Seek medical advice in 2-3 weeks		1617 (6.1)
	Safely manage at home		273 (1.0)

## Discussion

### Principal Results

This study is one of the first published studies of the triage recommendations of an AI-driven symptom checker chatbot generated by a US-based patient population. Over a 9-month period, we saw robust use, particularly from younger and female users. Just under half of the assessments were completed outside of typical physician office hours, suggesting that there is a significant number of low-acuity concerns for which tailored guidance is not easily accessible during off-hours.

Understanding the user demographics of a symptom checker tool is an important milestone before subsequent, more nuanced questions can be answered. For example, there is a recognized need to study whether the use of symptom checkers augments patients’ understanding and management of their illnesses, commonly described as “health literacy” [8]. Baseline health literacy, however, varies across patient demographics (including age) [11], and it must be taken into account when evaluating symptom checkers. Furthermore, health systems concerned about a widening “digital divide” driven by expanded virtual care options [12] rely on demographic information to identify and support patients who prefer to receive care through traditional channels.

This study is unique from previous work in that we assess the use of a symptom checker that has been deployed in partnership with a brick-and-mortar health system. Patient uncertainty about symptom checkers is recognized [13], and collaboration with a familiar health delivery mechanism could potentially improve patient engagement. To this end, our results show substantial use by older users (13.1% of users were aged 60 years and older), who are not typically considered to be heavy users of web-based tools. Furthermore, symptom checkers have been theorized to serve as surrogates for physician advice for patients who lack access to care [13]. Our population, however, is predominately part of the Sutter Health care network; thus, they are using the symptom checker in conjunction with available in-person care options.

### Comparison With Prior Work

A prior study of web-based symptom checkers found that users were predominantly female and had a mean age of 40 years [6]. This suggests that our symptom checker users are similar to users of other symptom checker tools, although skewing slightly younger. This younger age skew may be due to the promotion of the symptom checker chatbot by Sutter Health through predominately digital channels (eg, emails, website banners, digital newsletters), which may have created disproportionate awareness of the tool among patients who already use digital tools and are thus likely to be younger.

In previous studies, the triage recommendations of US-based nurse triage telephone lines reported high acuity recommendations in 19.7%-48.6% of calls versus 28.9% in the current study, medium acuity in 28%-48.2% of calls versus 50.9% in our study, and low acuity in 24%-36% of calls versus 20.1% in our study [14-16]. Accordingly, the distribution of the acuity of triage recommendations from the symptom checker chatbot appears to be generally comparable to that of US-based nurse triage telephone lines. These results are encouraging for the movement toward triage automation, which would enable the reallocation of clinicians to roles that better leverage their extensive training and would potentially improve health care staffing shortages.

### Limitations

Limitations of this study include a potential lack of applicability to other symptom checkers, given that the results are wholly dependent on the configuration of a single symptom checker. The results were also influenced by the interest in digital health tools of a single geographic population; thus, they may not be nationally generalizable. True appropriateness of the triage recommendations cannot be assessed without patient-level comparisons against the existing gold standard of clinician-staffed triage telephone lines, and further research is needed to evaluate the diagnostic accuracy of the tool. Finally, although access to the symptom checker was provided through the Sutter Health webpage, use of the checker did not require any login or verification of affiliation with the Sutter Health care network; therefore, non-Sutter Health patients may have been included in our results. However, this use by non-Sutter Health patients is thought to be minimal because the Ada Health symptom checker chatbot is freely available elsewhere on the web, and navigating through the Sutter Health webpage requires additional steps that are not likely to be taken by the general population.

### Conclusions

This study is one of the first published studies of the triage recommendations of an AI-driven symptom checker chatbot generated by a US-based patient population. Users of the chatbot were broadly representative of the general population of our region, although they skewed toward younger and female users. Our results suggest that the triage recommendations are acceptable; however, future research is needed to evaluate the medical accuracy of digital symptom assessment tools. While the recent emergence of COVID-19 and the need to take social distancing precautions may cause greater reliance on such tools, it is important to take an evidence-based approach to their deployment.

## Abbreviations

AI: artificial intelligence

## References

[1] Dieleman JL, Squires E, Bui AL, Campbell M, Chapin A, Hamavid H, Horst C, Li Z, Matyasz T, Reynolds A, Sadat N, Schneider MT, Murray CJL (2017). Factors Associated With Increases in US Health Care Spending, 1996-2013. JAMA.

[2] (2016). Global strategy on human resources for health: Workforce 2030. World Health Organization.

[3] Kocaballi AB, Berkovsky S, Quiroz JC, Laranjo L, Tong HL, Rezazadegan D, Briatore A, Coiera E (2019). The Personalization of Conversational Agents in Health Care: Systematic Review. J Med Internet Res.

[4] Espinoza J, Crown K, Kulkarni O (2020). A Guide to Chatbots for COVID-19 Screening at Pediatric Health Care Facilities. JMIR Public Health Surveill.

[5] Chambers D, Cantrell AJ, Johnson M, Preston L, Baxter SK, Booth A, Turner J (2019). Digital and online symptom checkers and health assessment/triage services for urgent health problems: systematic review. BMJ Open.

[6] Winn AN, Somai M, Fergestrom N, Crotty BH (2019). Association of Use of Online Symptom Checkers With Patients' Plans for Seeking Care. JAMA Netw Open.

[7] Semigran H, Linder J, Gidengil C, Mehrotra A (2015). Evaluation of symptom checkers for self diagnosis and triage: audit study. BMJ.

[8] Aboueid S, Liu RH, Desta BN, Chaurasia A, Ebrahim S (2019). The Use of Artificially Intelligent Self-Diagnosing Digital Platforms by the General Public: Scoping Review. JMIR Med Inform.

[9] Ada.

[10] Demographic Profile - California and Counties. State of California Employment Development Department.

[11] Baker DW, Gazmararian JA, Sudano J, Patterson M (2000). The association between age and health literacy among elderly persons. J Gerontol B Psychol Sci Soc Sci.

[12] Mullangi S, Kaushal R, Ibrahim SA (2019). Equity in the Age of Health Care Information Technology and Innovation. Med Care.

[13] Meyer AND, Giardina TD, Spitzmueller C, Shahid U, Scott TMT, Singh H (2020). Patient Perspectives on the Usefulness of an Artificial Intelligence-Assisted Symptom Checker: Cross-Sectional Survey Study. J Med Internet Res.

[14] Bogdan GM, Green JL, Swanson D, Gabow P, Dart RC (2004). Evaluating patient compliance with nurse advice line recommendations and the impact on healthcare costs. Am J Manag Care.

[15] Moore JD, Saywell RM, Thakker N, Jones TA (2002). An analysis of patient compliance with nurse recommendations from an after-hours call center. Am J Manag Care.

[16] O’Connell JM, Towles W, Yin M, Malakar CL (2016). Patient Decision Making: Use of and Adherence to Telephone-Based Nurse Triage Recommendations. Med Decis Making.

